# Metallocyclodextrins: Combining Cavitands with Metal Centres

**DOI:** 10.1002/open.201300033

**Published:** 2013-08-28

**Authors:** Rafael Gramage-Doria

**Affiliations:** [a] Van't Hoff Institute for Molecular Sciences, University of AmsterdamScience Park 904, 1098 XH, Amsterdam (The Netherlands) E-mail: r.gramagedoria@uva.nl

**Keywords:** cavitands, C–C bond formation, cyclodextrins, oschelation, phosphanes

**Awarding Institution:** Université de Strasbourg (France)

**Date Awarded:** January 3, 2012

**Supervisors:** Dr. Dominique Matt and Prof. Dr. Dominique Armspach, Université de Strasbourg (France)

In the last decades, combining the properties of a transition-metal ion with those of a covalently linked, molecular cavity has attracted a great deal of attention. Numerous studies focused on the design and synthesis of systems exploiting the binding properties of a receptor moiety attached to an active metal site, with the aim of mimicking nature’s metal-containing catalysts: metalloenzymes. Other potential applications involve (1) the study of metal-promoted reactions occurring in a confined environment in order to find out whether this strategy leads to selective transformations, and (2) molecular recognition via supramolecular interactions in conjunction with photo- or electrochemical applications. In this respect, cyclodextrins (CDs) and their chemically modified derivatives occupy a position of choice. Their rigid, conical structure, inherent chirality, as well as the presence of hydroxy groups that can be substituted in a regioselective manner, offer many possibilities in terms of cavitand (cavity-shaped ligand) design. So far, most studies have dealt with the smallest member of the family, namely α-CD, because of the ease with which it can be functionalised, β-CD derivatives remaining largely unexplored, even if their bigger inner space seems more appropriate for taking full advantage of the cavity. The present thesis is mainly concerned with the preparation of new *introverted* phosphanes (**WIDEPHOS** and **L**, Figure 1) derived from multifunctionalised methylated β-CDs and the study of their coordination and catalytic properties. The last part of the manuscript focuses on the use of a smaller α-CD analogue of the aforementioned phosphanes, namely the *trans*-chelating diphosphane **TRANSDIP** (Figure 1) in palladium-promoted carbon–carbon bond-forming reactions.

**Figure 1:**
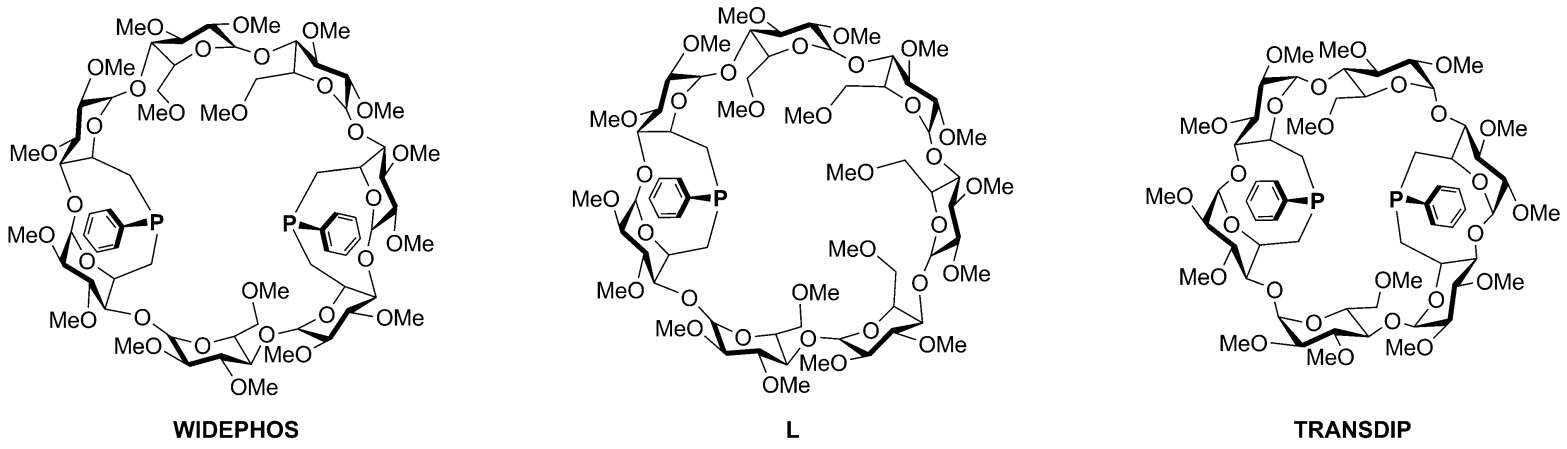
Cavitands designed and studied in this work.

The first part of this thesis focuses on reviewing transition-metal-based cavitands, for which the first and second metal coordination spheres are controlled by their cavity-shaped ligand. In the next part, four different regioselective double capping reactions were performed on α- and β-CD scaffolds. More precisely, the use of a rigid and bulky dialkylating reagent, containing two trityl-like moieties, gave access to an A,B,D,E-tetrafunctionalised β-CD regioisomer **1**, which is the key compound for preparing different types of cavitands bearing various functionalities, such as, disulfide **2**, diphosphane **WIDEPHOS** and disulfate **3** (Figure 2). A disulfate based on smaller α-CD (**4**, Figure 2) and introverted monophosphanes derived from β-CD were also reported. It is worth noting that disulfate-capped species **3** and **4** might be considered as potential precursors for the preparation of hetero-trifunctionalised cavitands. The capping of adjacent glucose units in CD-derived compounds turned out to be an extremely powerful strategy to straightforwardly prepare a library of cavitands.

**Figure 2:**
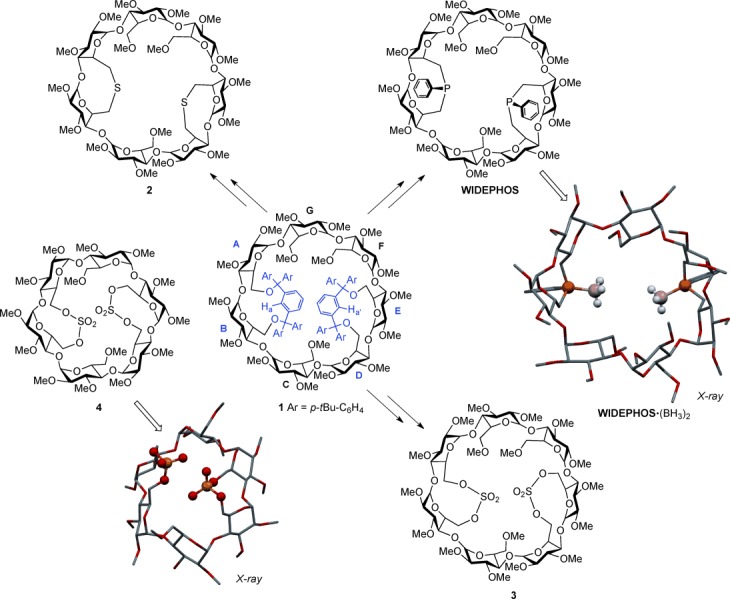
Different cavitands prepared from key compound **1**, showing the X-ray structure of **4** and adduct **WIDEPHOS⋅**(BH_3_)_2_.

The following chapters are devoted to study the coordination and catalytic properties of two new phosphane ligands built on a large β-CD scaffold: **WIDEPHOS** and **L. WIDEPHOS** bears two phosphorus lone pairs pointing to the cavity interior without being aligned because of the unsymmetrical nature of the β-CD scaffold. This geometrical feature, combined with the large distance separating the two phosphorus atoms (P⋅⋅⋅P distance of 6.91 Å in its corresponding phosphine oxide), promote the formation of “imperfect” *trans*-chelate complexes in which the metal centre swings about the ligand according to detailed NMR studies. This unprecedented molecular movement, named *oschelation*, allows each phosphorus atom to form an optimal bond in turn with the coordinated d^8^ (Pd^II^, Pt^II^, Rh^I^) or d^10^ (Au^I^) transition metal ions (Figure 3). This type of motion contrasts with the well-studied ligand hemilability, since no metal-phosphorus dissociation is observed in this case. Evidence for the weakness of one of the M–P bonds came from the facile preparation of unique palladium species **5** and **6** (Figure 3), in which one of the M–P bonds have been broken and non-conventional monophosphanes and P,O-chelating complexes form. Oschelation can be stopped by addition of a second metal centre to the cavitand. Indeed, further studies on **WIDEPHOS** proved that it is better suited for coordinating dinuclear fragments within the confinement of the large β-CD cavity. However, severe steric constraints on the metal first sphere of coordination result in the formation of unprecedented, single μ-chlorido-bridged dinuclear species; additionally stabilized by Cl⋅⋅⋅H hydrogen bonding between the intracavity-located chlorido ligands and inwardly oriented CD protons called H-5. In this new type of square-planar complexes, non-optimal orbital overlapping measured by the so-called tilt angle was also found to take place for one of the phosphorus atoms together with an oschelation movement involving nonidentical donor atoms, namely a phosphorus and an oxygen atom (Figure 3). Static gold(I) dinuclear complexes displaying similar imperfect orbital overlapping for one of the phosphorus atom were also prepared (Figure 3). **WIDEPHOS** was active in some palladium-catalysed reactions, although its performance was found to be poorer than **L** (Figure 1).

**Figure 3:**
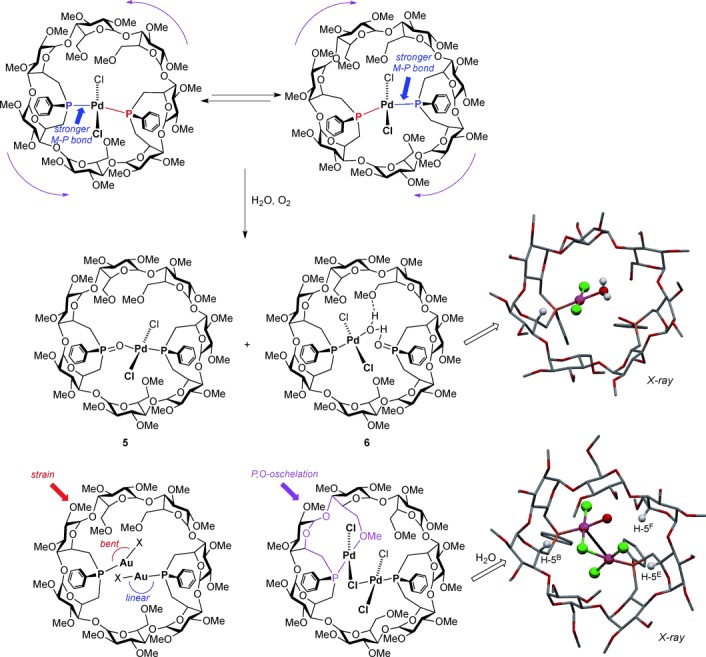
Balance-wheel movement of **WIDEPHOS** (top), reactivity of an *oschelate* palladium complex (middle) and rare dinuclear species derived from **WIDEPHOS** (bottom).

The last part of the manuscript concerns the unexpected C–C coupling reaction (ethane formation) mediated by a palladium *trans*-chelate complex derived from the previously reported diphosphane **TRANSDIP**. Several key intermediates involved in the mechanism of this C–C bond formation were isolated and identified (Scheme 1), notably the unprecedented κ^1^-*P*-[PdMe_3_(**TRANSDIP**)]Li complex (**9**). The confinement imposed by **TRANSDIP** on the palladium centre enabled direct visualisation of three types of highly reactive intermediates, in particular, of the first pentacoordinated alkyl complex containing the *trans*-spanning diphosphane **7**. In contrast to analogues based on other *trans*-chelating diphosphanes, the intermediate [PdMeCl-(**TRANSDIP**)] (**8**) undergoes dissociation of one of its phosphorus atoms upon nucleophilic attack of MeLi, re-coordination occurring after the reductive elimination step. Remarkably, the protecting role of the cavity towards M–Cl bonds is essential for stabilising a zerovalent palladium atom in one of the complexes (**10**). Finally, the perfect match between a [Pd(η^2^-O_2_)] moiety and the cyclodextrin core makes the [Pd(η^2^-O_2_)**TRANSDIP)**] complex (**11**) unusually stable compared with previously reported peroxo complexes.

**Scheme 1:**
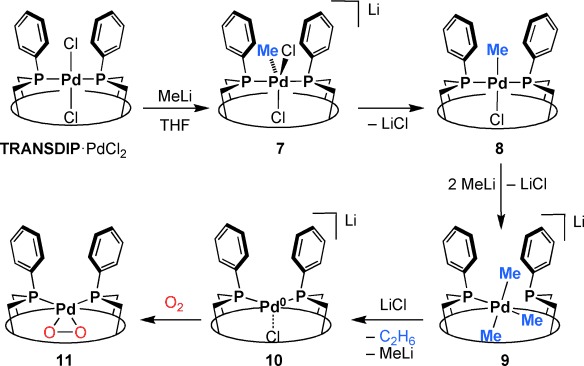
Following a C–C bond forming reaction on **TRANSDIP**.

Overall, the work described here emphasizes the importance of ligand design in building metalloreceptors derived from cyclodextrins. These new cavity-shaped molecules allowed the study of unprecedented dynamic coordination processes occurring in chelate complexes (*oschelation*), which proved to have a profound effect on metal reactivity. **WIDEPHOS**, with its two intra-annular, but wide apart donor atoms, constitutes a very peculiar cavitand allowing the formation of both mono and dinuclear complexes with extremely distorted coordination geometries. In particular, this study revealed that supramolecular interactions between a metal fragment and the cyclodextrin inner wall play a fundamental role in the stabilisation of otherwise unstable species. Lastly, we have shown the application of introverted phosphanes in homogeneous catalysis and the use of metallocavitands based on an authentic *trans*-spanning ligand (**TRANSDIP**) for unravelling, at that time unknown, mechanistic pathways associated with a palladium-promoted C–C bond formation. At this stage, one can anticipate that metallocavitands will create many opportunities regarding the catalytic outcome of reactions, in particular, in terms of selectivity that may result from the supramolecular confinement of non-conventional and otherwise unstable species and/or the activation of small molecules such as dioxygen, amongst others.

## Supplementary material

As a service to our authors and readers, this journal provides supporting information supplied by the authors. Such materials are peer reviewed and may be re-organized for online delivery, but are not copy-edited or typeset. Technical support issues arising from supporting information (other than missing files) should be addressed to the authors.
