# Supplementary material for: Metallocyclodextrins: Combining Cavitands with Metal Centres
Source: ChemistryOpen. 2013 Aug 28;2(5-6):176–9. doi: 10.1002/open.201300033 (PMC3892194; doi:10.1002/open.201300033)
Supplement: Supplementary file 1 [file open0002-0176-SD1.pdf]

Open Access

ChemistryOPEN
Thesis Treasury

Supporting Information

© 2013 The Authors. Published by Wiley-VCH Verlag GmbH & Co. KGaA, Weinheim

Metallocyclodextrins: Combining Cavitands with Metal Centres

Rafael Gramage-Doria*[a]

DOI: 10.1002/open.201300033

The full thesis passed by an examination board of the awarding institution can be found at

http://tel.archives-ouvertes.fr/docs/00/76/71/68/PDF/Gramage-Doria_Rafael_2012_ED222.pdf

Please note that Wiley-VCH is not responsible for the content or functionality of the full thesis supplied by the authors. Any queries (other than missing content) should be directed to the corresponding author for the article.

WILEY-VCH ChemPubSoc Europe
